# A case of anti-N-methyl-D-aspartate receptor encephalitis with associated ovarian teratoma in a 23-year-old female

**DOI:** 10.1016/j.radcr.2025.07.032

**Published:** 2025-08-05

**Authors:** Alexandra Hodder, Brandon Collins, Joseph Yang, Paul Jeon, Mark Stefanelli

**Affiliations:** a Faculty of Medicine, Memorial University of Newfoundland, 300 Prince Phillip Drive, St. John’s, NL, Canada, A1B 3V6; b Discipline of Radiology, Memorial University of Newfoundland, 300 Prince Phillip Drive, St. John’s, NL, Canada, A1B 3V6; c Discipline of Neurology, Memorial University of Newfoundland, 300 Prince Phillip Drive, St. John’s, NL, Canada, A1B 3V6

**Keywords:** Anti-NMDA receptor encephalitis, Encephalitis, Neuropsychiatric syndrome, Neuroimaging, Ovarian teratoma, Neuroradiology

## Abstract

Anti-N-methyl-D-aspartate (anti-NMDA) receptor encephalitis is an immune-mediated disease manifesting with a complex neuropsychiatric syndrome and anti-NMDA receptor antibodies within the cerebrospinal fluid. If not treated, it can progress to hypoventilation, coma, or death. It is often misdiagnosed as drug abuse, neuroleptic malignant syndrome, primary psychiatric disorder, or viral encephalitis. Despite its severity, the condition is highly responsive to treatment through decreasing antibody titers, and much of the potential morbidity can be avoided if it is recognized quickly. This case report describes a 23-year-old female from South Korea with no significant past medical history who presented to the emergency department with memory loss and migraine for 1 week. High levels of anti-NMDA receptors were identified. The diagnosis of anti-NMDA receptor encephalitis was further supported by computed tomography and magnetic resonance imaging findings of encephalitis and an ovarian teratoma, a common associated finding. Despite aggressive treatment with intravenous immunoglobulin, glucocorticoids and monoclonal antibodies, the patient failed to demonstrate a normal level of consciousness for the majority of the admission period and contracted numerous nosocomial infections. This case of a young, previously-well female outlines the need for early detection and treatment of this condition, considering the severe symptoms and deleterious outcomes. Its diagnosis relies on physicians having a high index of suspicion in young females presenting with acute psychiatric and neurological symptoms, as well as a focus on early appropriate radiological imaging. Further research is necessary on anti-NMDA-related conditions so that a timely, multidisciplinary approach to screening, diagnosis and treatment can be initiated.

## Introduction

Anti-N-methyl-D-aspartate (anti-NMDA) receptor encephalitis is a rare, immune-mediated form of limbic encephalitis characterized by neuropsychiatric symptoms and antibodies against the GluN1 subunit of the NMDA receptor in cerebrospinal fluid (CSF) [1]. It primarily affects young females, with a median age of onset of 22.6 years [1]. The condition has been associated with underlying neoplasms, particularly ovarian teratomas [1]. Early diagnosis is critical, as delayed treatment can result in significant morbidity or death; however, recognition remains challenging due to its variable presentation and overlapping features with primary psychiatric conditions. We have presented a case of anti-NMDA receptor encephalitis associated with ovarian teratoma in a young international student, highlighting diagnostic challenges, the importance of radiologic imaging in detection, and the need for multidisciplinary involvement to ensure timely intervention.

### Case report

A 23-year-old female originally from South Korea presented to a Canadian emergency department with a chief complaint of memory loss and migraine for approximately 1 week. The patient was an undergraduate international student on an exchange for the school semester. She endorsed ongoing confusion and difficulty with short-term memory. In addition, she demonstrated intermittent episodes of aggressive behaviors, including screaming, biting, and throwing water at the nursing stations in the emergency department. She possessed no past psychiatric history. Collateral history from an accompanying friend confirmed that the patient had been experiencing ongoing headaches for 2 weeks with minimal relief with over-the-counter medications.

Due to ongoing psychiatric symptoms, the patient was initially referred to psychiatry for a work-up of acute psychosis. However, the psychiatric team felt that her symptoms may be related to an organic cause, and neurology became involved to investigate for signs of potential causes such as encephalitis, seizure disorder, and meningitis. The patient’s physical examination demonstrated a normal pupillary response and neurological examination. No other concerning physical examination findings were noted. A lumbar puncture, computed tomography (CT) of the head, bloodwork panel, and electroencephalogram (EEG) were subsequently performed.

The patient’s head CT demonstrated no intracranial abnormalities, while the EEG demonstrated slowing and focal sharps over the left temporal region. Markers of autoimmune encephalitis, including CA19-9, carcinoembryonic antigen (CEA), alpha-fetoprotein (AFP), and CA125, were within normal limits. Urine toxicology screening was negative. A CSF sample obtained from the lumbar puncture indicated an elevated white blood cell count (29) with normal protein and glucose. The opening pressure was documented at 23 cm H2O. Additionally, high levels of anti-NMDA receptors were noted on the encephalitis antibody panel. In a young female, this raised concerns for anti-NMDA receptor autoimmune encephalitis, and the patient was subsequently admitted to neurology for further management.

**Fig. 1:**
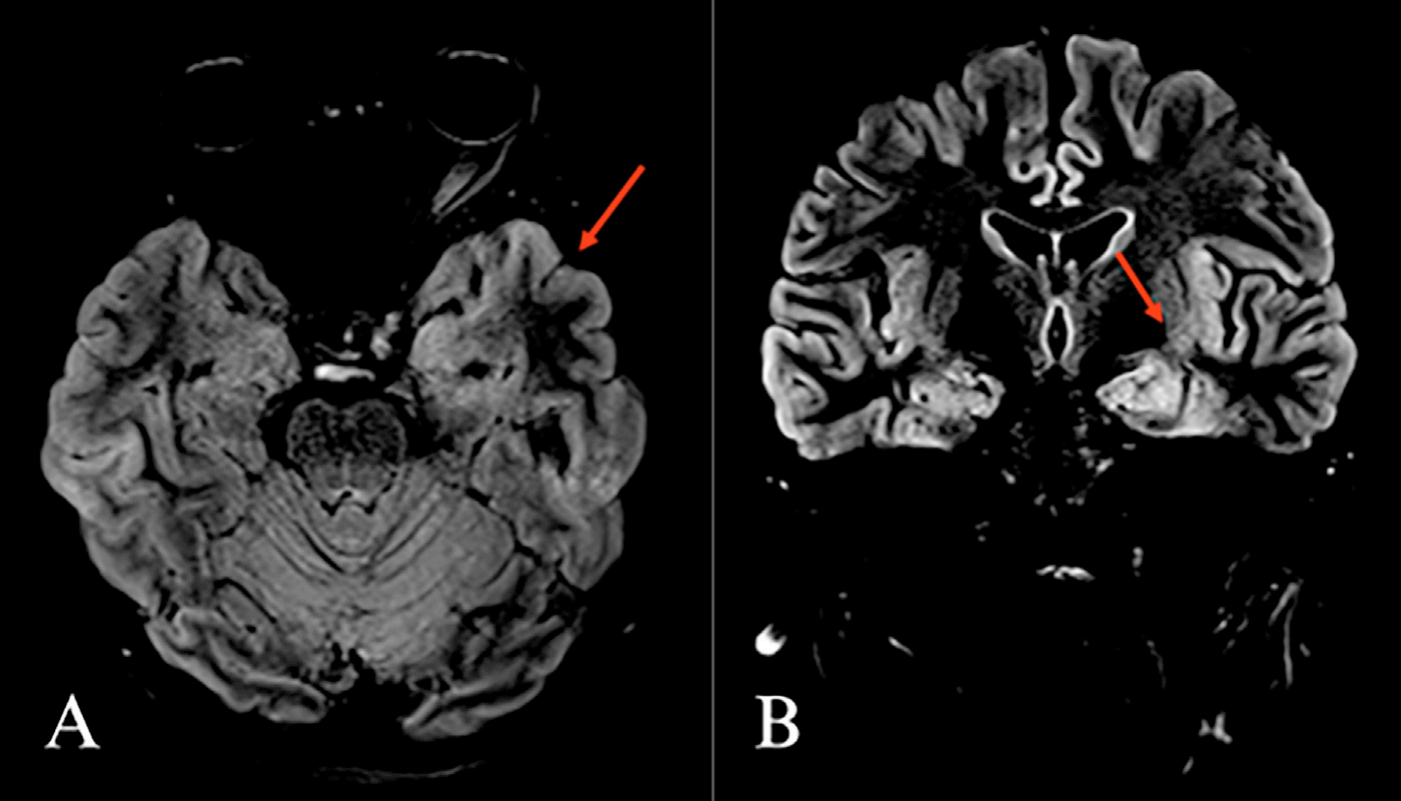
(A) T2-weighted fluid-attenuated inversion recovery (T2-FLAIR) magnetic resonance imaging (MRI) of the head shows subtle increased signal intensity (red arrows) along the inferior aspect of the left temporal lobe on axial and (B) coronal views.

Magnetic resonance imaging (MRI) of the head was obtained upon admission, which indicated a subtle increased signal intensity along the inferior aspect of the left temporal lobe on the T2-fluid-attenuated inversion recovery (FLAIR) images (see Fig. 1). Additionally, some increased signal intensity within the region of the insular cortex was visualized. Overall, the findings were suggestive of early encephalitis in keeping with the initial suspicion of anti-NMDA receptor autoimmune encephalitis.

**Fig. 2:**
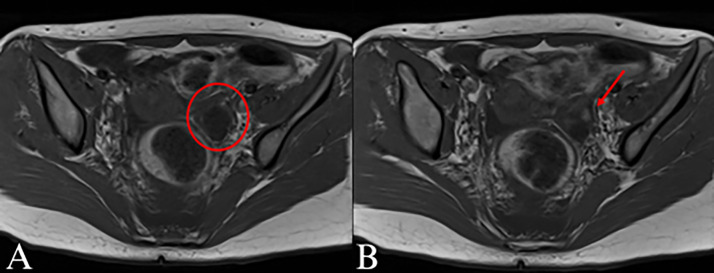
(A) Axial T1-weighted magnetic resonance imaging (MRI) of the pelvis shows the left ovary (red circle) (B) containing a hyperintense focus relative to surrounding tissue measuring up to 9mm (red arrow). In addition to an ovarian teratoma, the differential diagnosis of this could include a proteinaceous cyst, hemorrhage or fat.

**Fig. 3:**
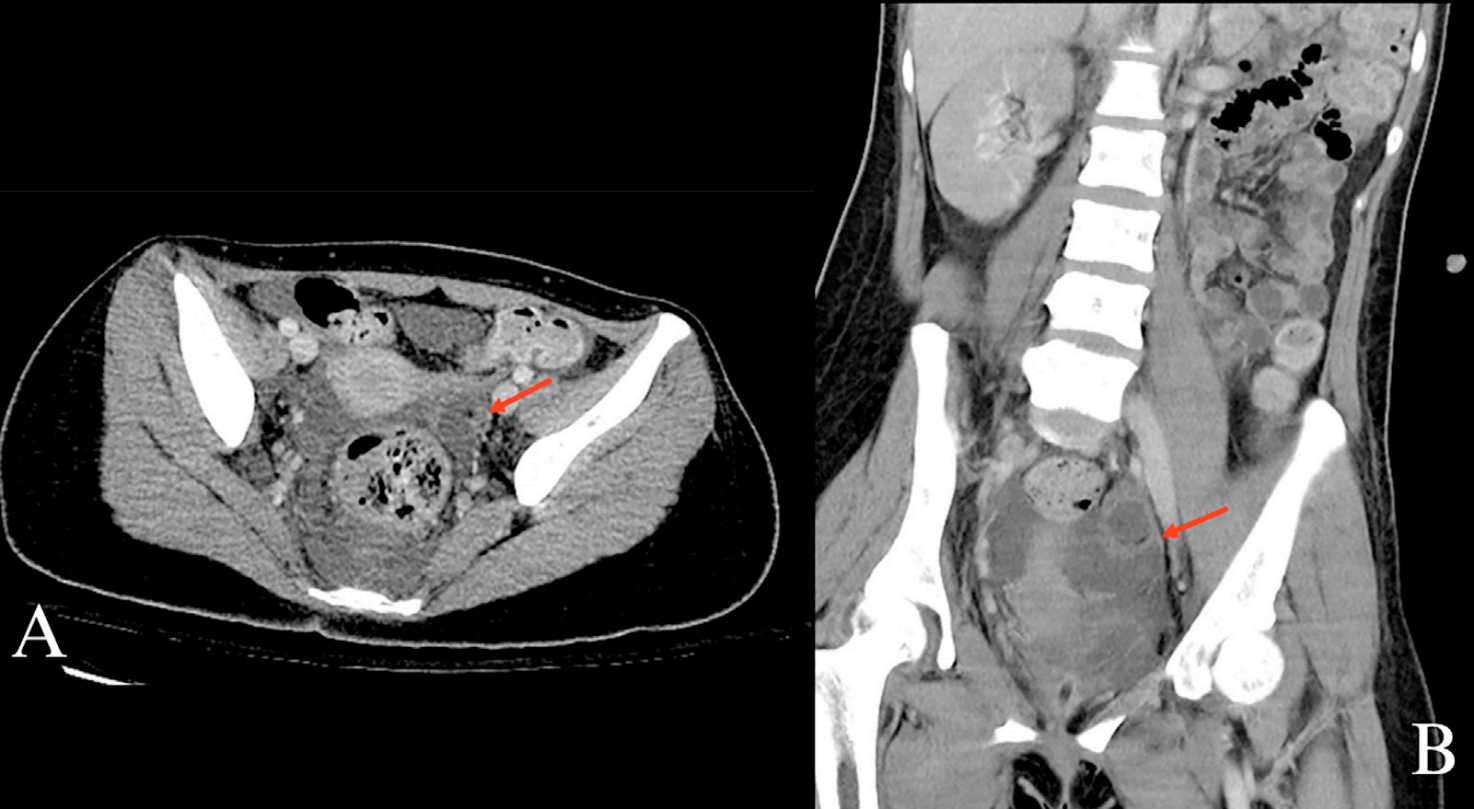
(A) Axial computed tomography (CT) of the abdomen and pelvis with contrast shows a left ovarian lesion (red arrow) measuring up to 2.7cm with a tiny focus of macroscopic fat in keeping with an ovarian teratoma. (B) Coronal CT of the abdomen and pelvis with contrast shows the same lesion (red arrow) as described above.

The presence of high levels of anti-NMDA antibodies warranted a search for an ovarian teratoma, as there is a strong correlation between these 2 entities [1]. Initially, an ultrasound of the pelvis was performed which questioned the presence of an ovarian teratoma. However, due to limited visualization of the uterus, a pelvic MRI was then performed. The pelvic MRI demonstrated a 9 mm by 7 mm lesion arising from the left ovary with possible presence of macroscopic fat (see Fig. 2). Gadolinium was not able to be administered and the entire MRI was unable to be completed due to patient agitation; thus, CT scan of the abdomen and pelvis was recommended. CT scan of the abdomen and pelvis performed with intravenous (IV) contrast demonstrated a left ovarian lesion measuring approximately 27 by 21 mm with a tiny hypodense focus of macroscopic fat, highly concerning for a teratoma (see Fig. 3). Other pertinent findings noted on this CT scan included bilateral pyelonephritis and right lower lung pneumonia, concerning for aspiration pneumonia (see Fig. 3).

The patient underwent a laparoscopic left ovarian cystectomy. During the surgery, a 2 cm ovarian cyst containing adipose tissue in keeping with a teratoma was identified. Due to its small size and intricate involvement of the left ovary, a left oophorectomy was also performed. The final pathology report indicated a diagnosis of a mature ovarian teratoma (dermoid cyst). The patient was initially treated with daily administration of intravenous immunoglobulin (IVIG) for 4 days, followed by methylprednisolone 1 mg/kg (50mg) IV daily for 16 days. These interventions yielded a limited response. The patient subsequently received 2 separate doses of rituximab 1g IV for 15 days with daily prednisone 50 mg via nasogastric (NG) tube. Between the 2 doses of rituximab, a 1-time dose of tocilizumab 8 mg/kg (344 mg) IV was administered. The patient failed to demonstrate a normal level of consciousness for the majority of the admission period and her reaction to stimuli was limited to spontaneous eye opening and orofacial dyskinesia.

**Fig. 4:**
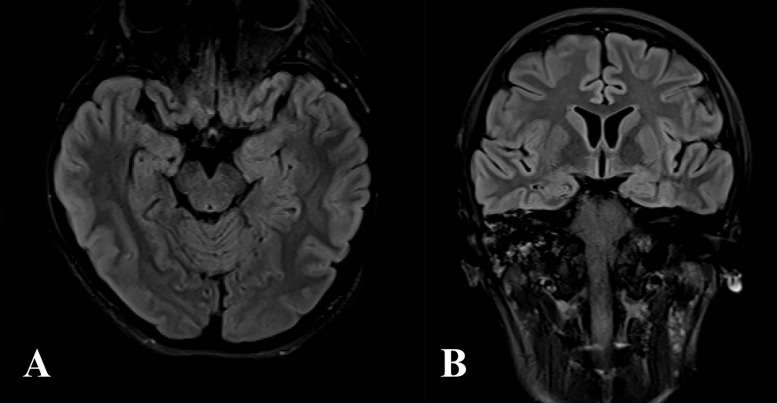
(A) T2-weighted magnetic resonance imaging (MRI) of the head at time of discharge, demonstrating improvement of the previously documented increased signal intensities along the inferior aspect of the left temporal lobe and the insular cortex on axial and (B) coronal views.

During the postoperative recovery period, the patient required placement of a tracheostomy and percutaneous endoscopic gastrostomy (PEG) tube with observation in the intensive care unit. Due to the immunosuppressive nature of rituximab and tocilizumab, the patient had multiple infections complicating her progress. At approximately 9 weeks of admission, the patient was deemed stable for transfer back to South Korea for further management. MRI of the head performed at the time of discharge demonstrated improvement of the previously documented increased signal intensities along the inferior aspect of the left temporal lobe and the insular cortex (see Fig. 4). The patient regained consciousness approximately 1 week after transfer to South Korea with gradual return of communication ability and memory. They displayed persistent anti-NMDA antibodies following discharge, requiring an additional course of rituximab, but eventually achieved a favorable clinical outcome with full recovery of language.

## Discussion

Anti-NMDA receptor encephalitis is an immune-mediated disease which manifests with a complex neuropsychiatric syndrome and the existence of GluN1 (a subunit of NMDA) antibodies within the CSF [1]. This case describes a young female who presented with acute neuropsychiatric symptoms and was ultimately diagnosed with anti-NMDA receptor encephalitis associated with an ovarian teratoma, based on radiological imaging. The patient underwent surgical resection of the teratoma and received immunotherapy, resulting in gradual clinical improvement.

This disease presents with a variety of possible symptoms that can be classified into psychiatric, cognitive, motor, autonomic, and nonspecific categories [1]. Characteristically, clinicians should be aware of patients with new onset of behavioral changes, psychosis, seizures, cognitive decline, and movement disorders. Anti-NMDA receptor encephalitis is a female-dominated disease with a 4:1 ratio of females to males affected [2]. 1.5 cases per 1 million people are expected per year [2]. The condition can have multiple etiologies, with the most common being infectious from herpes simplex virus (HSV) [1]. If not treated appropriately, eventual progression to hypoventilation and coma can occur [1]. Despite the severity of anti-NMDA receptor encephalitis (with 20% of patients developing focal deficits or dying from the condition), it is quite responsive to treatment through decreasing antibody titers [2]. However, the condition is often misdiagnosed as drug abuse, neuroleptic malignant syndrome, primary psychiatric disorder, or viral encephalitis [1]. An exclusion of recent herpes simplex virus and other causes of encephalitis must be made early in the diagnostic process. As well, on examination of the CSF, there must be a presence of IgG antibodies to the GluN1 receptor subunit [2].

Another category of etiology of this condition is from various tumors; benign ovarian mature teratomas are most common, but malignant transformation rarely occurs in 0.2%-2% of cases [3]. Malignant tumors are much less commonly associated with the condition, though these include small cell lung cancer, breast cancer, and lymphoma [3]. The age and sex of the patient dictates whether a clinical search for an ovarian teratoma is required. In approximately 50% of females with anti-NMDA receptor encephalitis older than 18 years, there were unilateral or bilateral ovarian teratomas; however, in females under 14 years, an ovarian teratoma was identified in only 9% of cases [1]. As was the case for this patient, those of Asian or African descent are more likely to have an associated teratoma [1]. The initial presentation of anti-NMDA receptor encephalitis with an associated ovarian teratoma can be challenging for clinicians, as these patients have more severe neurological symptoms as compared to those without an ovarian teratoma [1]. Identifying the presence of a teratoma may be difficult, as severe complications of anti-NMDA receptor encephalitis can occur even in very small teratomas [4].

Consequently, computed tomography (CT) and magnetic resonance imaging (MRI) have been utilized to visualize and characterize ovarian teratomas as they have excellent aptitude for distinguishing the fat contents characteristically seen in ovarian teratomas [5]. On CT, ovarian teratomas associated with anti-NMDA receptor encephalitis are smaller in size, have smaller amounts of fat, and are less likely to contain other components such as teeth or calcifications than ovarian teratomas in general [6]. On MRI, mature ovarian teratomas contain macroscopic fat and solid components, appearing hyperintense on T1-weighted imaging [6]. The presence of fat can be confirmed using T1-weighted fat-saturated sequences, which demonstrate signal dropout in fat-containing areas and heterogeneity if various germ cell layers are present [6]. Additionally, in-phase (IP) and out-of-phase (OOP) MRI gradient echo sequences are helpful in detecting small amounts of fat within these tumors. On OOP images, fat will also characteristically appear hypointense with the presence of India ink artifact at lipid-water interfaces [5].

Management of individuals with anti-NMDA receptor encephalitis associated with an ovarian teratoma should consist of immediate surgical removal of the teratoma and immunotherapy; this produces improved patient outcomes, a decreased rate of recurrence, and a quicker recovery time as compared to cases that do not undergo tumor excision [1].

To lessen the morbidity and mortality caused by the condition, a standardized diagnostic and screening algorithm may be developed. Most patients presenting with symptoms of anti-NMDA receptor encephalitis as described in existing case reports had undergone either or a combination of MRI, ultrasound, positron emission tomography (PET) or CT scans [1]. Some authors have recommended a transvaginal ultrasound as a first-line investigation for this purpose, given that it is more rapidly accessible [2,7]. While MRI has been shown to be effective, there is an overarching idea that the diagnosis is often missed when relying on this modality for detection of brain changes, since it may appear normal early in the disease course [1,8]. Some studies have suggested systematically screening young females for ovarian teratomas using abdominopelvic MRI, CT or transvaginal ultrasound; however, no specific screening intervals or protocols have been proposed or researched [2,7]. Other studies have advised first-line use of MRI with or without FDG-PET rather than CT due to the consideration of radiation exposure in young patients and particularly children, as well as the improved sensitivity offered by MRI [1].

The major strength of this case report is the multidisciplinary nature of the management of the case. Our patient, as mentioned, was initially consulted to psychiatry based on their symptoms. However, with a referral to neurology, it was able to be ascertained that their psychiatric symptoms were simply sequelae of the paraneoplastic ovarian teratoma. This is an excellent example to highlight the importance of multifaceted care and communication between various disciplines in medicine to produce a favorable patient outcome.

## Conclusion

Radiologists and other allied healthcare providers must be vigilant in their suspicion of anti-NMDA receptor encephalitis with associated ovarian teratoma in patients with a complex array of psychiatric and neurological symptoms. This case of a young, previously well female outlines the need for early detection and treatment of this condition in light of the severe symptoms and deleterious outcomes. The condition is highly responsive to treatment, and much of the potential associated morbidity can be avoided if it is recognized in a timely fashion. However, due to the nonspecific nature of the symptoms, it is commonly misdiagnosed. A multidisciplinary approach is essential to produce the best possible outcome for each patient, with a particular focus on early radiological imaging. Further research is necessary surrounding anti-NMDA-related conditions to increase awareness among healthcare organizations so that a timely approach to screening, diagnosis and treatment can be initiated.

## Patient consent

Complete written informed consent was obtained and documented from the patient in this case for the preparation of this manuscript and publication of its contents, as well as the associated images, data, and anonymized information.
